# Investigation of Factors and Psychological Diagnoses Associated with Cyberbullying/Victimization in Adolescents Applying to Child Psychiatry Clinic

**DOI:** 10.1192/j.eurpsy.2025.1117

**Published:** 2025-08-26

**Authors:** I. D. Çimen, A. M. Benk Ayaz, M. Dilli Gürkan, Z. Vatansever Pınar, B. Kardaş, Ö. Kardaş

**Affiliations:** 1 Kocaeli University, Kocaeli; 2 Tokat Dr. Cevdet Aykan Mental Health Hospital, Tokat; 3 Bandırma Research and Training Hospital, Balıkesir; 4 Freelancer, istanbul, Türkiye

## Abstract

**Introduction:**

The emergence of the internet and social media has not only changed the way people communicate, but has also brought with it a new type of bullying: cyberbullying. This type of bullying causes negative effects on the quality of life of individuals, especially adolescents and young adults, and becomes a serious problem for their mental health.

**Objectives:**

The aim of our study was to examine various factors that may be related to cyberbullying in adolescents applying to child psychiatry outpatient clinics and the psychiatric diagnoses of adolescents.

**Methods:**

A total of 174 patients aged 14-18 years who applied to the Department of Child Psychiatry and gave their consent to participate, were included in the study. The Cyberbullying Scale was applied to the youth, and the sociodemographic data form, cyberbullying study questions were applied to their parents.

**Results:**

The average age of the group was found to be 15.60±1.15. 122 (70%) participants were female and 52 (30%) participants were male. Of the 174 adolescents included in the study, 61 (35%) were found to be cybervictims, 29 (17%) were found to be cyberbullies, and 25 (14%) were found to be both victims and bullies. Cyber victimization was found to be significantly related to broken family type, mother’s exposure to cyberbullying on the internet, father’s internet usage time, and father’s exposure to cyberbullying on the internet. Cyberbullying was found to be significantly related to male gender, father’s low level of education, divorce, and mother’s and father’s internet usage time being between 0-1 hour/day. When the diagnoses of the patients were examined, 39 (22%) adolescents were diagnosed with neurodevelopmental disorders, 37 (21%) adolescents with mood disorders, and 68 (39%) adolescents with anxiety disorders. When the diagnosis groups were examined and the status of being a cyberbully or cybervictim, no significant difference was found in any diagnosis group. No significant relationship was observed between the adolescent’s diagnosis of any mental disorder and being a cyberbully or victim.

**Conclusions:**

Cyberbullying is not an issue to be taken lightly, and because it often co-occurs with traditional bullying, prevention and intervention programs need to address both contexts. In our study, it was determined that the majority of cyber victims were also cyberbullies. This situation shows the importance of evaluating bullying situations even though adolescents apply as victims in the clinic interviews. The results suggest that parents do not have enough knowledge about safe internet use and cannot control and guide their children properly. Therefore, during clinical interviews, cyberbullying/victimization issues should be discussed with mothers and fathers and how they can provide information to their children on this issue and safe internet use should be discussed.

**Disclosure of Interest:**

None Declared

